# Military tactical adaptive decision making during simulated military operational stress is influenced by personality, resilience, aerobic fitness, and neurocognitive function

**DOI:** 10.3389/fpsyg.2023.1102425

**Published:** 2023-02-08

**Authors:** Nicole M. Sekel, Meaghan E. Beckner, William R. Conkright, Alice D. LaGoy, Felix Proessl, Mita Lovalekar, Brian J. Martin, Leslie R. Jabloner, Alaska L. Beck, Shawn R. Eagle, Michael Dretsch, Peter G. Roma, Fabio Ferrarelli, Anne Germain, Shawn D. Flanagan, Christopher Connaboy, Amy J. Haufler, Bradley C. Nindl

**Affiliations:** ^1^Neuromuscular Research Laboratory, Warrior Human Performance Research Center, University of Pittsburgh, Pittsburgh, PA, United States; ^2^Military Sleep Tactics and Resilience Research Team, Department of Psychiatry, School of Medicine, University of Pittsburgh, Pittsburgh, PA, United States; ^3^U.S. Army Medical Research Directorate-West, Walter Reed Army Institute of Research, Joint Base Lewis-McChord, Washington, WA, United States; ^4^Behavioral Health and Performance Laboratory, Biomedical Research and Environmental Sciences Division, Human Health and Performance Directorate, KBR/NASA Johnson Space Center, Houston, TX, United States; ^5^Warfighter Performance Department, Operational Readiness and Health Directorate, Leidos/Naval Health Research Center, San Diego, CA, United States; ^6^Johns Hopkins University Applied Physics Laboratory, Laurel, MD, United States

**Keywords:** military personnel, decision making, resilience, psychological, exercise

## Abstract

Laboratory-based studies designed to mimic combat or military field training have consistently demonstrated deleterious effects on warfighter’s physical, cognitive, and emotional performance during simulated military operational stress (SMOS).

**Purpose:**

The present investigation sought to determine the impact of a 48-h simulated military operational stress (SMOS) on military tactical adaptive decision making, and the influence of select psychological, physical performance, cognitive, and physiological outcome measures on decision making performance.

**Methods:**

Male (*n* = 48, 26.2 ± 5.5 years, 177.7 ± 6.6 cm, 84.7 ± 14.1 kg.) subjects currently serving in the U.S. military were eligible to participate in this study. Eligible subjects completed a 96-h protocol that occurred over five consecutive days and four nights. Day 2 (D2) and day 3 (D3) consisted of 48-h of SMOS wherein sleep opportunity and caloric needs were reduced to 50%. Differences in SPEAR total block score from baseline to peak stress (D3 minus D1) were calculated to assess change in military tactical adaptive decision making and groups were stratified based on increase (high adaptors) or decrease (low adaptors) of the SPEAR change score.

**Results:**

Overall, military tactical decision-making declined 1.7% from D1 to D3 (*p* < 0.001). High adaptors reported significantly higher scores of aerobic capacity (*p* < 0.001), self-report resilience (*p* = 0.020), extroversion (*p* < 0.001), and conscientiousness (*p* < 0.001). at baseline compared to low adaptors, while low adaptors reported greater scores in Neuroticism (*p* < 0.001).

**Conclusion:**

The present findings suggest that service members whose adaptive decision making abilities improved throughout SMOS (i.e., high adaptors) demonstrated better baseline psychological/self-reported resilience and aerobic capacity. Further, changes in adaptive decision-making were distinct from those of lower order cognitive functions throughout SMOS exposure. With the transition of future military conflicts placing higher priority on enhancing and sustaining cognitive readiness and resiliency, data presented here demonstrates the importance of measuring and categorizing baseline measures inherent to military personnel, in order to change and train one’s ability to suffer less of a decline during high stress conditions.

## 1. Introduction

Warfare exposes military personnel to volatile, uncertain, complex, and ambiguous environments including sleep deprivation, caloric restriction, dehydration, prolonged bouts of physical exertion, and psychological burden (Nindl et al., 2018; Vikmoen et al., 2020; Conkright et al., 2021a). Laboratory-based studies designed to mimic combat or military field training through simulated military operational stress (SMOS) protocols have consistently demonstrated deleterious effects on warfighter’s physical, cognitive, and emotional performance/function (Hoyt et al., 2006; Vikmoen et al., 2020; Beckner et al., 2021a; Conkright et al., 2021a). In lieu of conducting cognitive research during live military scenarios due to operational security, and institutional human use review board-related constraints, surrogate efforts have been successful in mimicking operational stress in tightly controlled laboratory conditions and high-fidelity field and training exercises. In one such study, Lieberman et al. (2005) observed a 20% decrease in reaction time, 40% decrease in time to complete a memory task and deleterious effects in mental state and mood following an intense, 53-h, multifactorial military operational stress scenario among an elite group of U.S. Army soldiers. Similar findings resulted from a 72 h U.S. Navy SEAL training after which vigilance experienced a 37% decline with significant negative effects across mood domains and in working memory (Lieberman et al., 2002). LaGoy et al. (2022) examining daytime sleepiness and slow wave activity sleep under the present SMOS protocol, found that lower daytime sleepiness and lower absolute slow wave activity predicted better physical readiness at baseline but not changes in readiness. Further, sleep restriction has been strongly associated with cognitive and mood degradation, even in the absence of other stressors (Lieberman et al., 2002, 2005; LaGoy et al., 2021) but baseline characteristics such as aerobic fitness and resilience may buffer declines in vigilance during exposure to SMOS (Beckner et al., 2021a).

Other influential factors on military performance such as resilience, personality, mood state and physiology also contribute to the adaptive ability of the warfighter, referring to the ability to “engage, distinguish relevant from irrelevant information, consider options, and refine or create new commands in a timely manner and often under conditions of high stress” (Haufler et al., 2018). Whereas some innate personality traits and mood changes may benefit the Warfighter’s adaptive ability, others can be detrimental. Personal attributes may also distinguish successful soldiers as tactically competent. Neuroticism, defined by disproportional reactions characterized by negative emotions, including irritability, sadness, and worry, to frustrations and threat (Lahey, 2009), has been consistently associated with an increases the odds of attrition from basic training (Lee, 2010). Similarly, soldiers who exhibit more negative mood states (e.g., tension, fatigue, confusion) were less likely to meet qualification standards at the U.S. Army Ranger School (Tharion et al., 2013).

Select circulating biomarkers have been associated with performance and resilience in military personnel during operational stress (Beckner et al., 2021a,b; Conkright et al., 2021a). Higher concentrations of neuropeptide-Y (NPY) have been associated with greater mental alertness and were reported to increase in special forces soldiers undergoing military survival training; of note, NPY increases were positively correlated with cortisol (CORT; Morgan et al., 2000). Furthermore, brain-derived neurotropic factor (BDNF) is considered a key biomarker related to learning and memory (Miranda et al., 2019), while insulin-like growth factor-I (IGF-I) is neuroprotective and has importance for optimal cognitive health (Nindl et al., 2003, 2007; Nindl and Pierce, 2010). Thus, the collective assessment of NPY, CORT, BDNF, and IGF-I could inform about the adaptability of subjects undergoing SMOS.

Beckner et al. (2021a) in one of the first publications of the current study, presented the impact of SMOS on executive function as measured by a neurocognitive test consisting of 10 individual test batteries. Service members (SM) were stratified by tertiles: low (≤33.3rd percentile score), moderate (>33.3rd percentile and ≤66.7th percentile score), and high (>66.7th percentile score) based on trait resilience and aerobic fitness scores (Beckner et al., 2021a). Additionally, associations between executive function and neuroendocrine biomarkers that may contribute to cognitive performance before, during or after SMOS were examined. As a result, SMOS reduced SM vigilance by −11.3% and working memory by −5.6% but increased risk propensity by +9.5% with those SM exhibiting high aerobic fitness and high trait resilience demonstrating less of a decline. SMOS reduced circulating concentrations of all biomarkers except for oxytocin, which remained stable (Beckner et al., 2021a). Ultimately, these results demonstrate that high aerobic fitness and high trait resilience may buffer the impact of SMOS on vigilance. Although Beckner et al. (2021a) and others have quantified decrements in physical and core neurobehavioral functions under military operational stress, few have assessed how such decrements relate to more complex tactical military decision-making. Future military conflicts will place a higher priority on enhancing and sustaining cognitive readiness and resiliency, with current joint doctrine highlighting the need for warfighters to exhibit the ability to adapt and evolve to the dynamic environment and multi-domain battle of the contemporary operating theater, optimally resulting in more successful mission outcomes and critical to survivability (Cojocar, 2012). In an attempt to address this critical gap and to provide an ecologically valid and military-relevant assessment for tactical decision making, the Johns Hopkins University Applied Physics Laboratory was contracted by the Asymmetric Warfare Group (AWG) to conduct a study of soldier adaptability from which the Soldier Performance and Effective, Adaptable Response Task (SPEAR) was developed, tested and results of soldier adaptability were published (Haufler et al., 2018). The SPEAR task is a computer-based assessment that includes 18 trials of realistic military scenarios focused on security force assistance such as embassy relations, human rights issues, and training with native civilians and in-country cultural considerations. With limited time to do so, subjects were required to make an executive decision in response to the specific trial and adapt to the changing stimulus while retaining the overarching objectives of the block’s strategic context (Haufler et al., 2018). To date, the SPEAR task has exclusively been examined preliminarily in relation to biobehavioral correlates (e.g., heart period, respiratory sinus arrythmia, and skin conductance) and self-report measures of mental health (e.g., state–trait anxiety inventory, profile and Dispositional Resilience Scale; Haufler et al., 2018). This study therefore begins to fill such a gap by assessing the extent to which status in these various domains relates to specific military-decision making strategies. Specifically, the present investigation sought to determine the impact of a 48-h simulated military operational stress (SMOS) protocol/exposure on military tactical adaptive decision making, and the influence of selected psychological, physical performance, cognitive, and physiological outcome measures on decision making performance. We hypothesized that high adapting subjects, those with ability to improve in military relevant adaptive-decision making following 48-h of SMOS, would exhibit higher baseline measures of resiliency, aerobic fitness, and positive personality traits and concurrently, suffer less of a decline across select psychological, physical performance, cognitive, and physiological outcome measures.

## 2. Methods

### 2.1. Participants

Male (*n* = 48) subjects between the ages of 18 and 41 years old and currently serving in the U.S. military through Active Duty, Reserve, National Guard, or Reserve Officer Training Corps (ROTC) were eligible to participate in this study. Eligible subjects underwent service-specific physical fitness tests within the last year, reported a high level of comfort with shooting an M4/M16 weapon, had no current or recent (within last 3 months) injury that would prevent participation in sport or prevent deployment, and were not working shiftwork or taking medications known to affect sleep or cognitive performance. Individuals with current alcohol use disorder, a history of bipolar, psychotic, seizure, or neurological disorder were excluded. Individuals with a prior diagnosis of traumatic brain injury with current chronic post-concussive symptoms and rehabilitative treatment for traumatic brain injury, or suspected traumatic brain injury in the previous 6 months based upon the medical review of post-concussive symptoms were also excluded. Furthermore, individuals at high risk of obstructive sleep apnea without treatment were excluded. Total cohort participant characteristics are presented in Table 1. The study received approval from the Institutional Review Board at the University of Pittsburgh (STUDY19090271) and the U.S. Army Medical Research and Development Command’s Human Research Protection Office (HRPO). After completing a comprehensive telephone screening interview, eligible subjects were scheduled for an in-person consent process. Once subjects provided written, informed consent, a urine drug screening and breathalyzer test were conducted to confirm eligibility.

**Table 1:** Total cohort participant characteristics (*N* = 48).

Baseline variable	Mean ± *SD*
Age (yrs)	26.2 ± 5.5
Height (cm)	177.7 ± 6.6
Weight (kg)	84.7 ± 14.1
Total years of service	6.4 ± 4.8
DRRI2	20.3 ± 8.3
CD-RISC	84.5 ± 10.5
$\dot{V}$O_2peak_ (mL*kg*min^−1^)	41.1 ± 11.0

The Connor-Davison Resilience Scale (CD-RISC), the Deployment Risk and Resilience Inventory-II (DRRI-II), relative peak oxygen consumption (VO_2peak_).

### 2.2. Experimental procedures

Data presented herein are part of a larger study (U.S. Department of Defense award #W81XWH-17-2-0070; Beckner et al., 2021a; Conkright et al., 2021a,b). This prospective cohort study was designed to characterize the impact of 48 h of SMOS, involving sleep and caloric restriction and physical work, on military tactical adaptive decision making. Eligible subjects completed a 96-h protocol that occurred over five consecutive days and nights. The SMOS protocol has been previously described in detail (Beckner et al., 2021a; Conkright et al., 2021a). Briefly, subjects completed 1 day of familiarization testing (D0) followed by an adaptation night of uninterrupted sleep from 2,300 to 0700, that included sleep apnea testing. Night 0 was intended to familiarize subjects to the sleep environment and setup. After 1 day of baseline testing (D1), subjects were exposed to 48-h of SMOS (D2 and D3). During SMOS, subject’s sleep opportunity was reduced to 50% of the familiarization and baseline night sleep opportunities (0100–0300 and 0500–0700). Additionally, subjects received only 50% of their individual caloric needs, based on energy expenditure calculations using air displacement plethysmography (BodPod Body Composition System, Life Measurement Instruments, Concord, CA). On the evening of D3, subjects were allowed uninterrupted sleep from 2,300 to 0700. Following the night of recovery sleep, subjects completed the final day of testing (D4) and were dismissed at 1930. All meals were provided by the laboratory study team and consisted of a standard breakfast and “meals, ready to eat” (MRE). Water intake was tracked and provided by the study team *ad libitum*. Caffeine was prohibited during the study.

### 2.3. Military tactical adaptive decision-making assessment

The SPEAR task is a computer-based assessment of adaptive decision making during realistic military scenarios (Haufler et al., 2018). An abbreviated version of the task was utilized in the present study, though a detailed description of the entire SPEAR task has been previously reported (Haufler et al., 2018). Briefly, the task begins with instructions followed by a strategic context, mission statement, and commander’s intent, presented for 30–45 s each. Eighteen trials are then presented to closely approximate tactical challenges associated with the block’s strategic guidance, mission statement, and commander’s intent. Each trial begins with a fixation cross-presented for 3 s to alert the participant that the trial is beginning. This is followed by a scenario description (30 s), a video (30 s), a response prompt (reporting to higher headquarters, adjacent units, or subordinates; 10 s), and the response period (105 s) in which the subject types their plan of action in response to the challenge presented in the trial. The video presents a situation that is either consistent or inconsistent with the initial scenario description in order to introduce uncertainty and heighten adaptability. Consistent/inconsistent trials and direction of the response (provided in the prompt) is balanced within each block and presented in a pseudo-randomized order.

An abbreviated practice SPEAR task was administered on day 0 during familiarization testing in order to control for learning effects. Subjects completed all 18 trials on day 1 (baseline) and in a different order on day 3 (peak stress) within the same testing environment. Subject responses were subsequently graded by three trained, independent graders based on a scoring rubric that consisted of an eight-category scale in which each subcategory represented a different aspect of an adaptable response (Haufler et al., 2018). Subjects could earn one point for each category, with a range of possible scores per trial between zero and eight. If a score deviated by more than 3 points between graders, discussion, reconciliation, and rationale for an agreed upon trial score were written and used as the final grade. The responses were scored using the rubric which contained eight different aspects of an adaptable response (Haufler et al., 2018). The eight aspects are shown in Table 2. The results of the inter-rater reliability assessment for the 18 prompts measured on Day 1 and Day 3 are listed in Appendix A. Gwet’s AC estimates ranged from 0.95 to 0.98, almost perfect, according to the benchmarks for strength of agreement recommended by Landis and Koch (1977).

**Table 2:** Scoring rubric for tactical decision making (SPEAR) task created by Haufler et al. (2018).

Trials demonstrated	Identifies or recognizes altered situation	Takes action based on altered situation that is appropriate behavior for military (i.e., legal, ethical, moral)	Creates new approach—changes behavior—not doing the same thing. New approach is appropriate behavior for military (i.e., legal, ethical, moral)	Explains new approach application—how the new approach is implemented—includes breakdown of tasks	New approach meets commander’s intent. Focus is on purpose	New approach meets the trial-tactical situation and purpose	Explains how new approach meets commander’s intent, the purpose and shows the relationship	Explains how new approach meets the trial-tactical situation, the purpose and shows the relationship	Total score
									

Table illustrates the 8 aspects of SPEAR soldier adaptability task scoring. For decisions to qualify as adaptive, responses had to include the first three aspects: recognizing an altered situation, takes action based on the altered situation, and creates a new approach. Responses inclusive of additional aspects represent higher levels of soldier adaptive decision making (i.e., a higher SPEAR score is better adaptive decision making).

### 2.4. Psychological battery

The psychological test battery occurred following informed consent, prior to the familiarization day, and consisted of 3 questionnaires: the Connor-Davison Resilience Scale (CD-RISC), the NEO Personality Inventory Five Factor Inventory (NEO), and the Deployment Risk and Resilience Inventory-II (DRRI-II). The CD-RISC is a validated 25-item self-report measure that uses a 5-point Likert scale ranging from 0 to 4 to measure resilience, with higher scores indicative of higher resilience (Connor and Davidson, 2003). All 25 items are summed to derive the total CD-RISC score. The CD-RISC has demonstrated strong internal consistency among military cohorts and has been associated with retention during basic military training (Xie et al., 2016; Bezdjian et al., 2017; Beckner et al., 2021a). The NEO is a 240-item self-report that measures responses using a five-point Likert scale, ranging from 1 to 5: “strongly disagree,” “disagree,” “neutral,” “agree,” or “strongly agree.” NEO assesses five personality traits that are known to modulate reactivity to stressful experiences: extraversion, agreeableness, conscientiousness, neuroticism, and openness to experience, with higher scores within each of the five traits representing greater propensity toward that specific personality characteristic (Costa, 1992; Ashton, 2013). The Combat Experiences scale is one of the 17 scales within the DRRI-II, a self-report instrument that assesses different factors and domains that contribute to risk and resilience following military deployment. This scale measures exposure to combat-related circumstances (i.e., firing a weapon or being fired on, being attacked or witnessing an attack, encountering explosive devices etc.) with scores ranging from 17 to 102, with a higher score denoting more combat-related experiences (Vogt et al., 2013).

### 2.5. Aerobic fitness

Aerobic fitness was assessed using the Bruce protocol treadmill test (Froelicher et al., 1975). On the familiarization day, subjects completed the test on a Woodway treadmill to determine relative peak oxygen consumption (mL·kg^−1^·min^−1^) as measured using a metabolic cart (Parvo TrueOne® 2,400; Salt Lake City, UT).

### 2.6. Neurocognitive assessment

Neurocognitive assessments pertaining to this analysis included measures of risk propensity, emotion recognition, vigilant attention, short-term memory, and language-based logical reasoning. The Balloon Analog Risk Task (BART) quantifies risk propensity and impulsivity (Lejuez et al., 2002). The Emotion Recognition Test (ERT) assesses emotional identification through facial expressions (Basner et al., 2015). The Psychomotor Vigilance Test (PVT) assesses vigilant attention (Basner et al., 2015). An abbreviated (3-min), validated version of the PVT was used in this study (Basner et al., 2011). The BART, ERT, and PVT are from the *Cognition* test battery, a computerized task designed to assess cognitive function in high-performing adults and deemed most relevant to the present study (Basner et al., 2015). Subjects completed these tests each morning. Each afternoon after completion of a physical testing battery, subjects completed a neurocognitive test battery consisting of two tests: Match to Sample (MATCH) to assess short-term spatial memory (Shurtleff et al., 1994; Lieberman et al., 2002, 2014), and Grammatical Reasoning (GRAM) was adapted from the Baddeley Grammatical Reasoning Test (Baddeley, 1968) to evaluate language-based logical reasoning (Lieberman et al., 2005, 2014). Speed and accuracy scores were calculated for the BART, ERT, and PVT as previously described (Moore et al., 2017; Basner et al., 2020; Beckner et al., 2021a). GRAM and MATCH accuracy were calculated as the number of trials answered correctly throughout the assessment, per total number of trials completed with 24 total trials for MATCH and 32 for GRAM (Lieberman et al., 2002, 2014). Speed was calculated as reaction time to correct responses in seconds (Lieberman et al., 2002, 2014).

### 2.7. Mood states

The Profile of Mood States Questionnaire (POMS) was used to assess subjects’ mood each day at approximately the same time (0830). POMS is a validated psychological test consisting of a 65-item survey, wherein subjects current mood state according to 6 specific domains is categorized on a 5-point Likert scale (Curran et al., 1995). The six domains include: tension-anxiety, depression-dejection, anger-hostility, vigor-activity, fatigue-inertia, and confusion-bewilderment (McNair et al., 1971; Lieberman et al., 2005). Subjects responses were subsequently summed to determine a total mood disturbance (TMD) score, TMD is the sum of the negative scales plus the “non-positive” remainder of the positive Vigor-Activity scale such as that: Total Mood Disturbance = (anger and hostility + confusion and bewilderment + depression and dejection + fatigue and inertia + tension and anxiety) + (maximum vigor and activity score – actual vigor and activity score; McNair et al., 1971; Curran et al., 1995). Lower scores indicated a more positive mood state and conversely, higher scores indicating a more negative mood state (Conkright et al., 2021a).

### 2.8. Biological specimens

Subjects were fasted, and their blood was drawn from an upper extremity vein each morning (~08:00 h) *via* a 21- or 23-gauge needle (Becton, Dickinson and Company, Franklin Lakes, NJ). Using standard venipuncture procedures, blood was collected into appropriate collection tubes, EDTA for plasma (10 mL), SST for serum (10 mL), and P100 for protein preserved plasma (2 mL; Becton, Dickinson and Company, Franklin Lakes, NJ). SST tubes were allowed to clot at room temperature for 30 min prior to centrifugation, EDTA and P100 tubes were centrifuged immediately after collection. All tubes centrifuged at 1500 ×*g* for 15 min at 4°C (Beckner et al., 2021a). Blood supernatant was aliquoted and stored at −80°C for future analysis. Enzyme-linked immunoassays were conducted for each biomarker, using plasma samples from EDTA collection tubes for IGF-I (APLCO, Salem, United States), and BDNF MILLIPLEX Magnetic Bead Panel 3 (EMD Millipore, Burlington, Massachusetts). plasma obtained from P100 tubes for NPY analysis (R&D Systems, Minneapolis, MN, United States), and serum samples for cortisol (Alpco, Salem, United States). Sensitivity for each assay was as follows: IGF-I: 0.09 ng/ML; BDNF: 10 pg./mL, cortisol: 0.4 μg/dL. Sensitivity information was not available for NPY. All samples were measured in duplicate with intra-assay coefficients of variation of 10% or less.

### 2.9. Statistical analysis

A total of three trained raters participated in evaluating each subject completed SPEAR trials. Performance was evaluated on an ordinal scale (0–8) with 8 representing the best possible score, per trial. In order to account for high percent agreement, the Gwet’s AC coefficient was calculated (Landis and Koch, 1977; Gwet, 2008; Gwet, 2014). Disagreement among raters was weighted using ordinal weights. Statistical analysis for the inter-rater reliability was conducted using Stata/SE 16 (StataCorp LLC; College Station, TX).

Differences in SPEAR score from baseline to peak stress (D3 minus D1) were calculated to assess change in military tactical adaptive decision making that may occur following 48-h SMOS. The sample was divided into low adaptors (SPEAR total block score decrease from baseline to peak stress; *N* = 20) and high adaptors (SPEAR total block score increase from baseline to peak stress; *N* = 28). Subjects whose scores remained the same were excluded from analysis (SPEAR total block score change of 0 from baseline to peak stress; *N* = 3). Independent samples t tests were performed to analyze differences in baseline measures (i.e., CD-RISC, NEO, DRRI-II, aerobic fitness) between groups. Two-way mixed-measures ANOVAs were performed to analyze the interaction of group (low or high adaptors) and day (D1, baseline or D3, peak stress) on each domain (i.e., neurocognitive, mood state, neuroendocrine). If the two-way interaction was not significant, main effects of group and of day were reported and interpreted. If assumptions for ANOVAs were not met, logarithmic (LN), square root (SQRT), and reciprocal (REC) data transformations were conducted. Successful data transformations were performed for the following variables: LN basal BDNF, REC basal cortisol, REC IGF-I, SQRT years of service, SQRT ERT speed, SQRT PVT speed, LN BART speed. If assumptions for normality were not met (i.e., Shapiro–Wilk *p* < 0.05), nonparametric tests were performed. All analyses were performed using IBM SPSS Statistics for Mac, version 26 (IBM Corp., Armonk, NY). Alpha was set at 0.05 *a priori* for all analyses and Bonferroni-adjusted for multiple pairwise comparisons. Effect size *via* partial eta-squared$\;\left(\eta_{p}^{2}\right)$ was calculated from ANOVA results.

## 3. Results

Subjects consumed an average of 2,306 ± 592.0 kcals on D0, 2,555 ± 494.5 kcals on D1 and 1,441 ± 265.8 kcals on D4. During SMOS, subjects consumed 1,536 ± 254.7 kcals on D2 and 1,579 ± 275.9 kcals on D3. There was a significant difference between groups in total caloric intake during unrestricted days such that high adaptors consumed more calories than did low adaptors (2,611.2 ± 491.3 vs. 2,328.8 ± 418.7, respectively, *p* = 0.04, $\eta_{p}^{2}$ = −0.61). Body mass declined by 1.2 kg over the study period from 85.7 ± 13.9 kg on D0 to 84.5 ± 13.5 kg on D4. While this represented a significant change in the total cohort (*p* = 0.04, $\eta_{p}^{2}$ = 0.07), there was not a significant change in body mass between groups. At baseline, subjects slept for an average of 7.3 ± 0.4 h on night 1 (baseline) and 7.5 ± 0.2 h on night 4 (recovery). Over the stressor, subjects averaged just 3.7 ± 0.2 h on night 2 and 3.8 ± 0.1 h on night 3. There was a significant difference in unrestricted sleep between groups, such that high adaptors slept more than did low adaptors (7.5 h. ± 0.2 vs. 7.4 h. ± 0.3, respectively, *p* = 0.04, $\eta_{p}^{2}$ = −0.70). Differences in baseline characteristics are listed in Table 3.

**Table 3:** Participant characteristics (*N* = 48) of low adaptors (*n* = 20) and high adaptors (*n* = 28).

Baseline variable	Low adaptors, mean ± *SD*	High adaptors, mean ± *SD*
Age (years)	26.05 ± 5.15	26.50 ± 5.97
Height (cm)	177.27 ± 5.67	178.48 ± 7.13
Weight (kg)	83.13 ± 14.67	86.92 ± 13.95
Years of service	6.12 ± 5.49	6.81 ± 4.45
DRRI-II	19.30 ± 4.50	21.36 ± 10.43
CD-RISC	80.20 ± 11.15	87.71 ± 9.66*
VO_2peak_ (mL*kg*min^–1^)	30.40 ± 4.67	48.38 ± 8.23*

Data presented as mean ± SD. *denotes significant group differences at baseline from Significance was set at *p* < 0.05. DDRI-II, Deployment Risk and Resilience Inventory-II; CD-RISC, Connor Davidson Resilience Scale.

### 3.1. Military tactical adaptability performance

Overall, military tactical decision-making adaptability remained stable throughout the operational stress scenario, from D1 (69.0 ± 18.3) to D3 (68.1 ± 21.1). Low adaptors (*n* = 20) exhibited an average score of 69 ± 17 on D1 and 56 ± 21 on D3, a statistically significant mean decrease of −12.50 ± 12.56 (*p* < 0.001, $\eta_{p}^{2}$ = 0.530). High adaptors (*n* = 28) also scored an average of 69 ± 19, but then improved on D3 with an average score of 76 ± 17, a statistically significant mean increase of 7.32 ± 6.27, *p* < 0.001, $\eta_{p}^{2}$ = 0.530.

### 3.2. Baseline psychological battery based on military tactical adaptability

**Figure 1:**
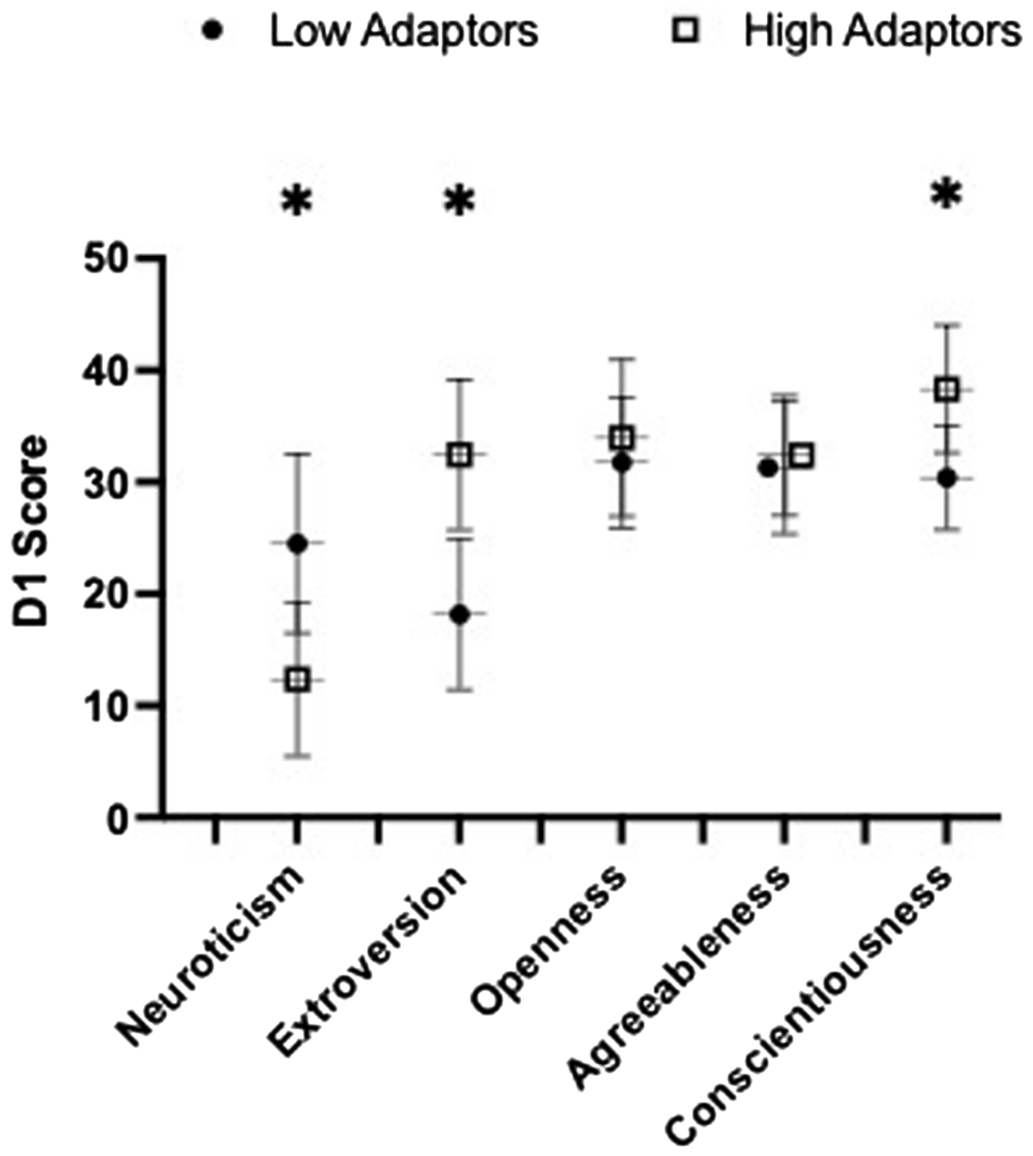
Baseline (D1) the NEO Personality Inventory Five Factor Inventory (NEO) personality scores stratified by low and high adaptive groups are presented here. Data presented as group mean ± SD. Significance was set at *p* < 0.05. 6 edges denotes significant group differences.

High adaptors reported significantly higher scores of self-reported resilience at baseline compared to low adaptors (87.7 ± 9.7 vs. 80.2 ± 11.2, *p* = 0.020, $\eta_{p}^{2}$ = 0.119). Low adaptors reported greater scores in neuroticism compared to high adaptors (12.4 ± 6.9 vs. 24.5 ± 8.0; *p* < 0.001, $\eta_{p}^{2}$ = 0.401) and conversely, high adaptors were more extroverted (32.4 ± 6.7 vs. 18.2 ± 6.7, *p* < 0.001, $\eta_{p}^{2}$ = 0.534) and conscientious (38.3 ± 5.7 vs. 30.4 ± 4.7, *p* < 0.001, $\eta_{p}^{2}$ = 0.362) than low adaptors (Figure 1). Groups did not differ in openness or agreeableness.

### 3.3. Aerobic fitness based on military tactical adaptability

Groups significantly differed at baseline aerobic fitness testing such that high adaptors had a significantly higher aerobic capacity compared with low adaptors (48.4 ± 8.2 vs. 30.4 ± 4. 7 mL/kg/min, respectively; *p* < 0.001, $\eta_{p}^{2}$ = 0.627).

### 3.4. Neurocognitive performance during SMOS based on military tactical adaptability

There was a main effect of day for multiple neurocognitive tests including those testing for language-based reasoning, vigilant attention, and emotion recognition (Table 4). GRAM speed was statistically significantly different on D1 (M = 3.6, SD = 0.9) compared to D3 (M = 2.8, SD = 1.2), *p* < 0.001, $\eta_{p}^{2}$ = 0.328, a mean decrease of −0.11.

**Table 4:** Neurocognitive test batteries stratified by low and high adaptivity at baseline and peak stress.

Variable	Group	Day 1: Baseline	Day 3: Peak Stress	Interaction effect, *p* (partial *η^2^*)	Main effect of group, *p* (partial *η^2^*)	Main effect of day, *p* (partial *η^2^*)
MATCH Speed (s)	High	4.9 ± 1.6	4.4 ± 1.8	0.823 (0.001)	0.526 (0.010)	0.121 (0.058)
	Low	4.9 ± 1.7	4.8 ± 1.9			
MATCH Accuracy	High	16 ± 4.0	15 ± 4.0	0.776 (0.002)	0.192 (0.041)	0.062 (0.082)
	Low	14 ± 5.0	13 ± 5.0			
GRAM Speed (s)	High	3.6 ± 1.0	2.9 ± 1.1	0.735 (0.003)	0.630 (0.006)	**<0.001 (0.328)**
	Low	3.6 ± 1.0	2.7 ± 1.4			
GRAM Accuracy	High	25 ± 5.0	24 ± 6.0	0.386 (0.018)	0.202 (0.038)	0.177 (0.043)
	Low	23 ± 6.0	22 ± 5.0			

Data presented as mean ± SD across baseline and peak stress and presented as p (partial η^2^) for group comparisons. Significance was set at *p* < 0.05. MATCH, Match to sample; GRAM, Grammatical Reasonings. Bold denotes significant finding.

ERT accuracy decreased from D1 (M = 71.6, SD = 11.0) compared to D3 (M = 66.3, SD = 16.0), *p* = 0.026, $\eta_{p}^{2}$ = 0.103, a mean decrease of −0.052. PVT slowness increased from D1 (M = 2.4, SD = 0.1) compared to D3 (M = 2.5, SD = 0.1), *p* = < 0.001, $\eta_{p}^{2}$ = 0.311, a mean increase of 0.03. PVT accuracy increased from D1 (M = 0.9, SD = 0.1) compared to D3 (M = 0.8, SD = 0.2), *p* < 0.001, $\eta_{p}^{2}$ = 0.308, a mean increase of 0.03. There were no main effects of day or group for risk propensity (BART), or short-term memory (MATCH; Table 5).

**Table 5:** Cognition test batteries stratified by low and high adaptability at baseline and peak stress.

Variable	Group	Day 1: Baseline	Day 3: Peak stress	Interaction effect, *p* (partial *η^2^*)	Main effect of group, *p* (partial *η^2^*)	Main effect of day, *p* (partial *η^2^*)
PVT slowness (10-[1/RT])	High	2.4 ± 0.1	2.43 ± 0.1	0.332 (0.020)	0.336 (0.020)	**<0.001 (0.311)**
	Low	2.4 ± 0.1	2.49 ± 0.2			
PVT accuracy (%)	High	91 ± 15%	83 ± 21%	0.200 (0.035)	0.104 (0.056)	**<0.001 (0.308)**
	Low	86 ± 13%	72 ± 22%			
BART speed (ms)	High	7.3 ± 0.4	7.3 ± 0.5	0.645 (0.005)	0.273 (0.027)	0.881 (0.001)
	Low	7.5 ± 0.7	7.5 ± 0.5			
BART accuracy (%)	High	59 ± 15%	60 ± 22%	0.280 (0.025)	0.370 (0.018)	0.177 (0.039)
	Low	51 ± 18%	59 ± 19%			
ERT speed (ms)	High	45.0 ± 6.1	42.5 ± 7.2	0.996 (0.000)	0.071 (0.069)	0.197 (0.036)
	Low	48.8 ± 8.6	46.3 ± 15.5			
ERT accuracy (%)	High	74.1 ± 8.5%	68.4 ± 11.2%	0.826 (0.001)	0.096 (0.059)	**0.026 (0.103)**
	Low	68.1 ± 12.7%	63.4 ± 21.3%			

Data presented as mean ± SD across baseline and peak stress and presented as p (partial η^2^) for group comparisons. Significance was set at *p* < 0.05. PVT, Psychomotor Vigilance Test; BART, The Balloon Analog Risk Task; ERT, Emotion Recognition Test. Bold denotes significant finding.

**Figure 2:**
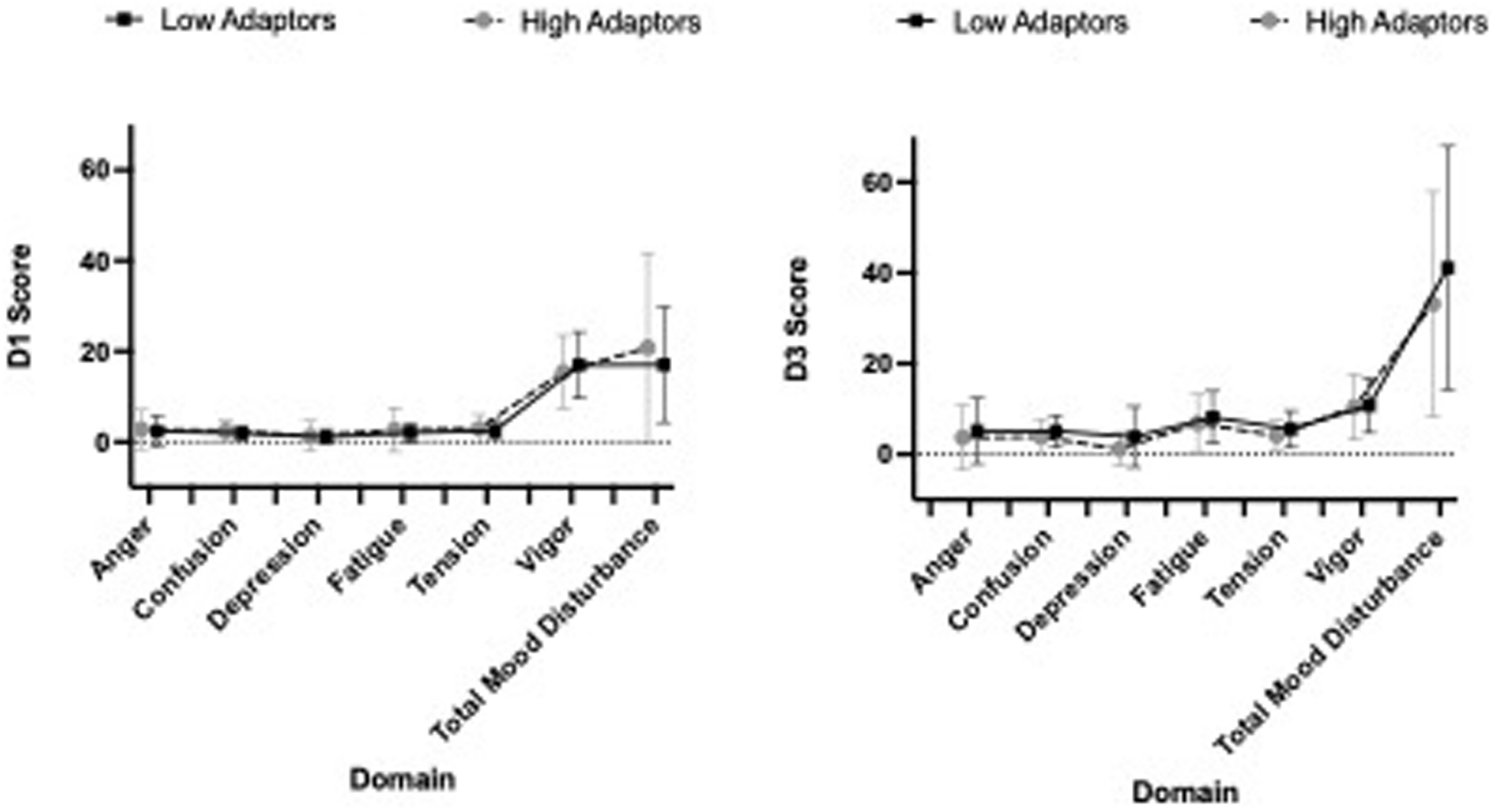
Profile of Mood States (POMS) scores stratified by low and high adaptive groups are presented in Figure 2. Baseline scores (D1) are presented on the left, while D3 (peak stress) scores are presented on the right.

Tension, fatigue, confusion and total mood disturbance all significantly increased from D1 to D3 (all *p*-values < 0.001), while vigor decreased (*p* < 0.001). Depression and anger did not significantly change over the course of the stressor (Figure 2). There were no main effects of group for POMS.

### 3.5. Physiological response to 48-h SMOS based on military tactical adaptability

Natural logarithmic transformations corrected for non-normality in resting IGF-I showed significantly different concentrations on D1 (M = 36.6, SD = 13.1) compared to D3 (M = 33.6, SD = 11.2), *p* = 0.026, $\eta_{p}^{2}$ = 0.110, a mean decrease of-2.94. Reciprocal transformations corrected for non-normality in resting NPY and cortisol but did not yield significant differences by day or group. Natural log transformations corrected for non-normality in resting BDNF, and similarly did not yield significant differences (Table 6).

**Table 6:** Blood biomarkers stratified by low and high adaptability at baseline and peak stress.

Variable	Group	Day 1: Baseline	Day 3: Peak stress	Interaction effect, *p* (partial *η^2^*)	Main effect of group, *p* (partial *η^2^*)	Main effect of day, *p* (partial *η^2^*)
IGF-I (nmol/L)	High	39.3 ± 14.4	36.2 ± 11.9	0.891 (0.000)	0.058 (0.081)	**0.026 (0.110)**
	Low	32.6 ± 9.9	29.8 ± 9.0			
BDNF (pg/mL)	High	4,137 ± 4,886	4,145 ± 3,959	0.266 (0.028)	0.316 (0.023)	0.203 (0.037)
	Low	4,460 ± 4,526	7,444 ± 10,058			
CORT (μg/dL)	High	27 ± 0.1	28 ± 0.1	0.665 (0.005)	0.798 (0.002)	0.170 (0.045)
	Low	27 ± 0.1	27 ± 0.1			
NPY (pg/mL)	High	9,560 ± 16,404	8,592 ± 14,362	0.411 (0.023)	0.773 (0.003)	0.083 (0.097)
	Low	7,555 ± 17,689	7,202 ± 17,617			

1 Raw data shown for reader clarity. Data presented as mean ± *SD* across baseline and peak stress and presented as p (partial η^2^) for group comparisons. Significance was set at *p* < 0.05. NPY, neuropeptide-Y; CORT, cortisol; BDNF, Brain-derived neurotropic factor; IGF-I, Insulin-like growth factor-I. Bold denotes significant finding.

## 4. Discussion

High adaptors—those with ability to improve in military relevant adaptive-decision making following 48-h of SMOS including decreased sleep opportunity, caloric restriction and physical work exhibited greater positive personality traits, self-report resilience and aerobic fitness compared to low adaptors. However, and contrary to our hypothesis, neurocognitive performance declined similarly in low and high adaptors following 48-h of SMOS, though grammatical reasoning speed improved in both groups. Similarly, neuroendocrine biomarkers were minimally impacted, with the exception of modest declines in IGF-I. Collectively, the present findings suggest that trait resilience, positive personality traits, and aerobic capacity were higher at baseline in subjects whose adaptive decision-making improved during SMOS (i.e., high adaptors).

### 4.1. High adaptors exhibited greater positive personality traits, self-report resilience, and aerobic fitness than did low adaptors

High adaptors exhibited significantly less neuroticism, and greater conscientiousness and extroversion than low adaptors. This finding is supported by prior literature examining the role of personality in military leadership and in group effectiveness, wherein low neuroticism and high conscientiousness best predicted high leadership effectiveness in the Australian Military Officer Training School (McCormack and Mellor, 2002). Conscientiousness, including such personal characteristics as order, achievement striving and dependability (Callister et al., 1999; Halfhill et al., 2005), positively aligns with the traits necessary to be successful in the simultaneous perception, cognition, and action aspects demanded in the adaptive decision making task (Haufler et al., 2018). Further, the high extroversion displayed in high adaptors may have caused these subjects to be more engaged in the group dynamics of the study protocol, theoretically acting as a buffer from deficits during SMOS. This theory aligns with the widely studied benefits of social coherence (McCraty, 2017; Salas et al., 2017). In contrast, neuroticism, including such personal characteristics as impulsivity and vulnerability has been inversely associated with success in military cohorts (Callister et al., 1999; Paunonen et al., 2006; Lee, 2010) and positively correlates with mental health problems in a large cohort of Chinese medical school students (Peng et al., 2012).

High adaptors scored significantly higher on the CD-RISC test compared to low adaptors. The CD-RISC, with higher scores indicative of higher resilience, assesses the ability to adapt to challenging circumstances (Connor and Davidson, 2003), a trait paramount to warfighters. As described by Haufler et al. (2018) military service members must consistently respond to multifaceted and competing demands within the modern warfare operational setting. Further, they must be able to acutely distinguish relevant from irrelevant information, consider their options, and adapt their trajectory in a timely fashion often under conditions of severe stress (Haufler et al., 2018). Following 6-months of U.S. Air Force basic training, the CD-RISC scale successfully predicted early unsuitability attrition (e.g., mental health or behavioral adjustment difficulties), revealing those who remained active exhibited higher CD-RISC scores, than did the separated trainees (Bezdjian et al., 2017). Further, resiliency—as measured by the CD-RISC scale—was found to be a prominent moderating factor between negative life events and mental health issues (Peng et al., 2012).

High adaptors demonstrated significantly greater aerobic capacity than did low adaptors. It is well recognized that a high level of physical fitness, specifically aerobic fitness, is necessary for success in military duties, including deployment and occupation (Kyröläinen et al., 2018). High aerobic capacity has obvious physical performance benefits, including elevated mission-specific performance, reduction of injury risk (Taylor et al., 2008; Lisman et al., 2013), and enhanced recovery from high-intensity exercise (Hoffman, 1997) with limited recovery time (Ojanen et al., 2018; Conkright et al., 2021a). Further, higher aerobic fitness has been shown to improve cognition (e.g., reasoning, planning, and problem solving; Stern et al., 2019) and is positively associated with working memory, (Bunce and Murden, 2006) greater task accuracy, and improved reaction time, and sleep quality (Taheri and Valayi, 2018; Lefferts et al., 2019). In a large community based clinical trial, greater aerobic capacity was universally found to be highly associated with greater executive function, a relationship that became more pronounced with age (Stern et al., 2019). Similarly, within the same experimental protocol, Beckner et al. (2021a) found that greater aerobic fitness and greater resiliency were protective in buffering the impact of operational stress on vigilant attention. High adaptors demonstrated high aerobic capacity prior to undergoing SMOS, a key mediator to offset the inevitable detriment in physical capacity sustained during stress.

### 4.2. SMOS negatively impacted vigilance response time, vigilance accuracy, and emotional recognition but had minimal impact on reasoning

After sustaining 48-h of SMOS, high and low adaptors experienced similar changes in neurocognitive function. The speed at which the correct response was given in the language-based reasoning battery improved similarly across low and high adaptors while vigilant attention speed declined. This finding is consistent with prior literature demonstrating impaired reaction time and vigilance following simulated stress (Lieberman et al., 2005; Vrijkotte et al., 2016; Beckner et al., 2021a). Vigilant attention contributes to the readiness to respond to relevant stimuli, a profoundly important function during the volatility of warfare. Prior literature supports the notion that the processes underlying social and emotional cognition suffer detriment under stressful conditions, but there have been mixed results pertinent to the effect of stress on facially-oriented emotional recognition. Some studies have demonstrated the positive, or even compensatory reaction to acute stress resulting in greater attention to emotional cues, which is thought to be synchronized with the evolutionary framework suggesting that the ability to detect and assess potential threats under adverse conditions is essential to human survival (Barel and Cohen, 2018). In contrast, others have found no such enhancement (Li et al., 2014). An integral part of the adaptive decision making, the basis on which the groups were stratified, is the necessity for Warfighters to effectively interact and engage with locals from communities that do not share their personal, religious, or cultural beliefs and customs. As Oden et al. (2015) describes, these differences induce uncertainty into an already chaotic and intense operational environment, during which an abrupt, unchecked response can catalyze a violent, sometimes unnecessary, escalation. Response time during the grammatical reasoning test was not impaired over the course of the stressor, as both high and low adaptors improved from baseline to peak stress. This is in contrast to prior findings as others have observed between a 7%–15% decline in grammatical reasoning number correct after 53–60 h of sleep deprivation (Lieberman et al., 2005) and deterioration in performance in even elite Special Forces soldiers, following acute stress (Kallinen and Ojanen, 2021).

### 4.3. SMOS negatively impacted mood state and IGF-I with high and low adaptors responding similarly

During a 5-day protocol including 48-h of SMOS, high and low adaptors experienced similar changes in mood state and neuroendocrine biomarkers. High and low adaptors experienced similar changes in negative mood states including significant increases in tension, fatigue, confusion, and total mood disturbance while suffering a decline in vigor. When Harris et al. assessed the cognitive function and psychological state prior to and 1-week post a Navy Survival, Evasion, Resistance, and Escape School—a period of physical stress, sleep and caloric deprivation and psychological distress—similar deteriorations in vigor were found, as well as increased fatigue and confusion (Harris et al., 2005). Similarly, in another study examining negative mood states in male Marines after completion of high-altitude military training, Marines’ experienced significant changes in fatigue and vigor post training, with levels that did not significantly differ from adult male psychiatric outpatients (Bardwell et al., 2005). IGF-I has consistently shown sensitivity to military operational stress (Nindl et al., 2003; Henning et al., 2011; Vikmoen et al., 2020; Beckner et al., 2021b) and was the only neuroendocrine biomarker to decline by −8.3% during SMOS, while BDNF, cortisol and NPY remained stable. Caloric restriction—particularly energy and protein restriction—have been shown to exert strong influences on IGF-I (Nindl and Pierce, 2010). In the present study, subjects energy and protein declined by 49 and 48%, respectively on restricted (D2, D3) vs. unrestricted days (D0, D1, D4).

### 4.4. Limitations

The present study has several limitations that should be considered. While this study simulated stressors that typically occur in military settings, namely physical work, sleep and caloric restriction, there were no real-life consequences for poor performance. Despite explicit instruction to perform best effort across all domains, non-compliance and limited effort may be a potential confounding factor. The present study lacked a control group and thus, despite attempting to control for learning effects by way of multiple test exposures, limited definitive discrimination. Future studies should consider control group inclusion as to further eliminate potential extraneous variables. Furthermore, men and women respond to stress differently (Chaplin et al., 2008; van den Bos et al., 2009), such that these results cannot be extrapolated to female subjects. Though the impact of each individual stressor cannot be ascertained, the present study provides an ecologically valid assessment of the impact of SMOS on tactical decision-making and how it relates to psychological factors, physical fitness, mood, and neurocognitive performance.

## 5. Conclusion

High adaptors (i.e., those with ability to improve in military relevant adaptive-decision making following 48-h of SMOS that included physical work, and sleep and caloric deprivation), distinguished themselves from low adaptors with greater baseline resiliency, positive personality traits including greater extroversion and conscientiousness, and higher aerobic fitness. Conversely, high and low adaptors exhibited similar responses in neurocognitive function, included measures of risk propensity, emotion recognition, vigilant attention, short-term memory, and language-based logical reasoning, and neuroendocrine biomarkers. While prior studies have quantified the physical and cognitive decrements that occur during exposure to military operational stress, few have assessed how that relates to tactical military decision making. With the transition of future military conflicts placing higher priority on enhancing and sustaining cognitive readiness and resiliency, data presented here demonstrates the importance of measuring and categorizing baseline measures inherent to military personnel, in order to change and train one’s ability to suffer less of a decline in tactical decision-making capabilities during stress. Further, the magnitude of real-world multi-stressor environments within the contemporary operating theater will often exceed those simulated during laboratory study. Therefore, the deleterious effects of SMOS on tactical decision making, vigilance response time and accuracy, emotional recognition and mood present here may exponentially worsen during the realities of military conflict with the ultimate liability of catastrophic injury or death. With between 80% and 85% of military accidents resulting from decreased cognitive performance (Thomas and Russo, 2007), it is imperative to not just mission success but the survivability of Warfighters to appraise and evaluate the decrements found here and install the necessary countermeasures to buffer the inevitable decline caused by military operational stress.

## Data availability statement

The raw data supporting the conclusions of this article will be made available by the authors, without undue reservation.

## Ethics statement

The studies involving human participants were reviewed and approved by Institutional Review Board at the University of Pittsburgh U.S. Army Medical Research and Development Command’s Human Research Protection Office (HRPO). The patients/participants provided their written informed consent to participate in this study.

## Author contributions

BCN, SDF, CC, AJH, ML, AG, MD, PGR, and FF contributed to the conception and design of the study. NMS, MEB, WRC, ADL, FP, BJM, LRJ, ALB, and SRE collected the data, analyzed the data, and organized the database. NMS and ML performed the statistical analysis. NMS wrote the first draft of the manuscript. All authors contributed to the article and approved the submitted version.

## Funding

PGR was supported in part by KBR’s Human Health and Performance Contract NNJ15HK11B through the National Aeronautics and Space Administration.

## Conflict of interest

The authors declare that the research was conducted in the absence of any commercial or financial relationships that could be construed as a potential conflict of interest.

## Publisher’s note

All claims expressed in this article are solely those of the authors and do not necessarily represent those of their affiliated organizations, or those of the publisher, the editors and the reviewers. Any product that may be evaluated in this article, or claim that may be made by its manufacturer, is not guaranteed or endorsed by the publisher.

## Author disclaimer

The authors of this report are entirely responsible for its content. Any opinions, findings, and conclusions or recommendations expressed in this material are those of the authors, are not to be construed as official, and do not necessarily reflect those of the US Government, the Department of Defense, the Department of the Army, the National Aeronautics and Space Administration, the Department of the Navy, KBR, or Leidos. This material has been reviewed by the Walter Reed Army Institute of Research. There is no objection to its presentation and/or publication. The investigators have adhered to the policies for protection of human subjects as prescribed in AR 70–25.
