# Supplementary material for: Military tactical adaptive decision making during simulated military operational stress is influenced by personality, resilience, aerobic fitness, and neurocognitive function
Source: Front Psychol. 2023 Feb 8;14:1102425. doi: 10.3389/fpsyg.2023.1102425 (PMC9944034; doi:10.3389/fpsyg.2023.1102425)
Supplement: Supplementary file 1 [file Table_1.DOCX]

**Military Tactical Adaptive Decision Making During Simulated Military Operational Stress Is Influenced By Personality, Resilience, Aerobic Fitness, and Neurocognitive Function**

Nicole M. Sekel^1^, Meaghan E. Beckner^1^, William R. Conkright^1^, Alice D. LaGoy^1,4^, Felix Proessel^1^, Mita Lovalekar^1^, Brian J. Martin^1^, Leslie R. Jabloner^1^, Alaska L. Beck^1^, Shawn R. Eagle^1^, Michael Dretsch^5^, Peter G. Roma^2,6^, Fabio Ferrarelli^4^, Anne Germain^4^, Shawn D. Flanagan^1^, Christopher Connaboy^1^, Amy Haufler^3^, Bradley C. Nindl^1^

**Supplementary Material:**

**Appendix A:** Inter-rater reliability assessment for the 18 SPEAR task prompts

| **Prompt** | **Day 1** | | | **Day 3** | | |
| --- | --- | --- | --- | --- | --- | --- |
|  | **% agreement** | **Gwet's AC (95% CI)** | ***p* value** | **% agreement** | **Gwet's AC (95% CI)** | ***p* value** |
| Prompt # 1 | 0.9702 | 0.9101 (0.8746, 0.9455) | 0.000 | 0.9744 | 0.9328 (0.8947, 0.9708) | 0.000 |
| Prompt # 2 | 0.9714 | 0.9288 (0.9062, 0.9514) | 0.000 | 0.9661 | 0.8871 (0.8566, 0.9176) | 0.000 |
| Prompt # 3 | 0.9548 | 0.8480 (0.8021, 0.8938) | 0.000 | 0.9620 | 0.9013 (0.8625, 0.9402) | 0.000 |
| Prompt # 4 | 0.9848 | 0.9731 (0.9590, 0.9872) | 0.000 | 0.9641 | 0.8791 (0.8412, 0.9171) | 0.000 |
| Prompt # 5 | 0.9624 | 0.8735 (0.8395, 0.9075) | 0.000 | 0.9615 | 0.8600 (0.8123, 0.9076) | 0.000 |
| Prompt # 6 | 0.9557 | 0.8508 (0.8035, 0.8981) | 0.000 | 0.9664 | 0.9002 (0.8689, 0.9316) | 0.000 |
| Prompt # 7 | 0.9648 | 0.8989 (0.8645, 0.9332) | 0.000 | 0.9682 | 0.9211 (0.8938, 0.9483) | 0.000 |
| Prompt # 8 | 0.9772 | 0.9380 (0.9133, 0.9626) | 0.000 | 0.9563 | 0.8432 (0.8083, 0.8780) | 0.000 |
| Prompt # 9 | 0.9775 | 0.9445 (0.9165, 0.9725) | 0.000 | 0.9658 | 0.8843 (0.8579, 0.9108) | 0.000 |
| Prompt # 10 | 0.9699 | 0.9259 (0.8946, 0.9573) | 0.000 | 0.9722 | 0.9035 (0.8760, 0.9310) | 0.000 |
| Prompt # 11 | 0.9624 | 0.8866 (0.8449, 0.9283) | 0.000 | 0.9778 | 0.9414 (0.9133, 0.9694) | 0.000 |
| Prompt # 12 | 0.9631 | 0.8719 (0.8339, 0.9099) | 0.000 | 0.9712 | 0.9326 (0.9024, 0.9629) | 0.000 |
| Prompt # 13 | 0.9590 | 0.8888 (0.8444, 0.9333) | 0.000 | 0.9746 | 0.9208 (0.8836, 0.9581) | 0.000 |
| Prompt # 14 | 0.9604 | 0.8604 (0.8260, 0.8947) | 0.000 | 0.9736 | 0.9289 (0.8978, 0.9601) | 0.000 |
| Prompt # 15 | 0.9724 | 0.9033 (0.8690, 0.9375) | 0.000 | 0.9541 | 0.8608 (0.8154, 0.9061) | 0.000 |
| Prompt # 16 | 0.9590 | 0.8652 (0.8308, 0.8996) | 0.000 | 0.9756 | 0.9214 (0.8899, 0.9528) | 0.000 |
| Prompt # 17 | 0.9620 | 0.9046 (0.8657, 0.9436) | 0.000 | 0.9640 | 0.8878 (0.8308, 0.9448) | 0.000 |
| Prompt # 18 | 0.9736 | 0.9146 (0.8816, 0.9476) | 0.000 | 0.9665 | 0.8903 (0.8490, 0.9315) | 0.000 |
